# Effectiveness and Biomechanical Analysis of the Ortho-Bridge System on Femoral Fracture Healing: A Retrospective Analysis

**DOI:** 10.1007/s43465-022-00687-4

**Published:** 2022-08-09

**Authors:** Yubin Qi, Lin Yao, Yuntao Long, Guilai Zuo, Qingjie Zhang, Zhenlin Liu, Wen Wang

**Affiliations:** 1 Shandong Key Laboratory of Rheumatic Disease and Translational Medicine, Department of Orthopedic Surgery, The First Affiliated Hospital of Shandong First Medical University & Shandong Provincial Qianfoshan Hospital, No. 16766, Jingshi Road, Jinan, Shandong China; 2 Tianjin Walkman Biomaterial Co., Ltd. Newton Laboratory, No. 19 Technology Road, South District, Jinghai Economic Development Zone, Jinghai Country, Tianjin, China; 3 Jinxiang Affiliated Hospital of Jining Medical College, No.117, East Jinfeng Road, Shandong Jining, China

**Keywords:** Ortho-Bridge System, Locking compression plate, Femoral shaft fracture, Primary fracture healing, Secondary fracture healing, Trauma, Intramedullary nailing fixation, Internal plate fixation, Biomechanical analysis, Retrospective

## Abstract

**Background:**

Among the surgical methods for femoral fractures, the Ortho-Bridge System (OBS) appears to heal fractures via an uncommon process. We compared its effectiveness and biomechanical aspects to those of a locking compression plate (LCP) and explained the healing process demonstrated by the OBS.

**Methods:**

Eleven femoral shaft fracture cases treated with OBS between July 2017 and May 2020 were retrospectively reviewed. Clinical and radiographic data were collected during regular postoperative follow-up visits and assessed via the Harris Hip Score and Knee Society Score. We performed biomechanical experiments of OBS. We simulated different fracture conditions and selected appropriate screw holes at the fracture’s far and near segments. The OBS module was placed according to the position of LCP’s locking hole at both ends of the fracture; then, a static three-point bending test was performed.

**Results:**

All patients had contralateral callus growth with secondary fracture healing. Healing time was 3–5 months with excellent hip and knee function. When the key screw distance was 22–34 mm, the OBS was significantly less stiff than the LCP (*P* < 0.05). The stiffness of LCP and OBS decreased significantly when the key screw distance was 49–82 mm, with the LCP being slightly stronger (*P* < 0.05).

**Conclusions:**

Femoral shaft fracture treatment with OBS demonstrated secondary healing. When the distance between the key screws was 20–40 mm, the elasticity was higher in OBS than in LCP, possibly producing axial micro-motion to stimulate callus formation and promote fracture healing, which differ from the plate’s primary healing process.

## Introduction

The femoral shaft is a common fracture site in trauma patients [1]. According to epidemiological statistics, femoral fracture occurs in a typical bimodal age distribution and most commonly among young adult men with high-energy injury mechanisms. Proximal femoral fractures are more common in postmenopausal elderly women, who are prone to accidental walking injuries [2]. There have been many surgical methods employed for femoral fractures, including anterograde and retrograde intramedullary nails, various types of steel plates, and external fixators. However, each treatment method has its own advantages and disadvantages [3–8].

**Fig. 1:**
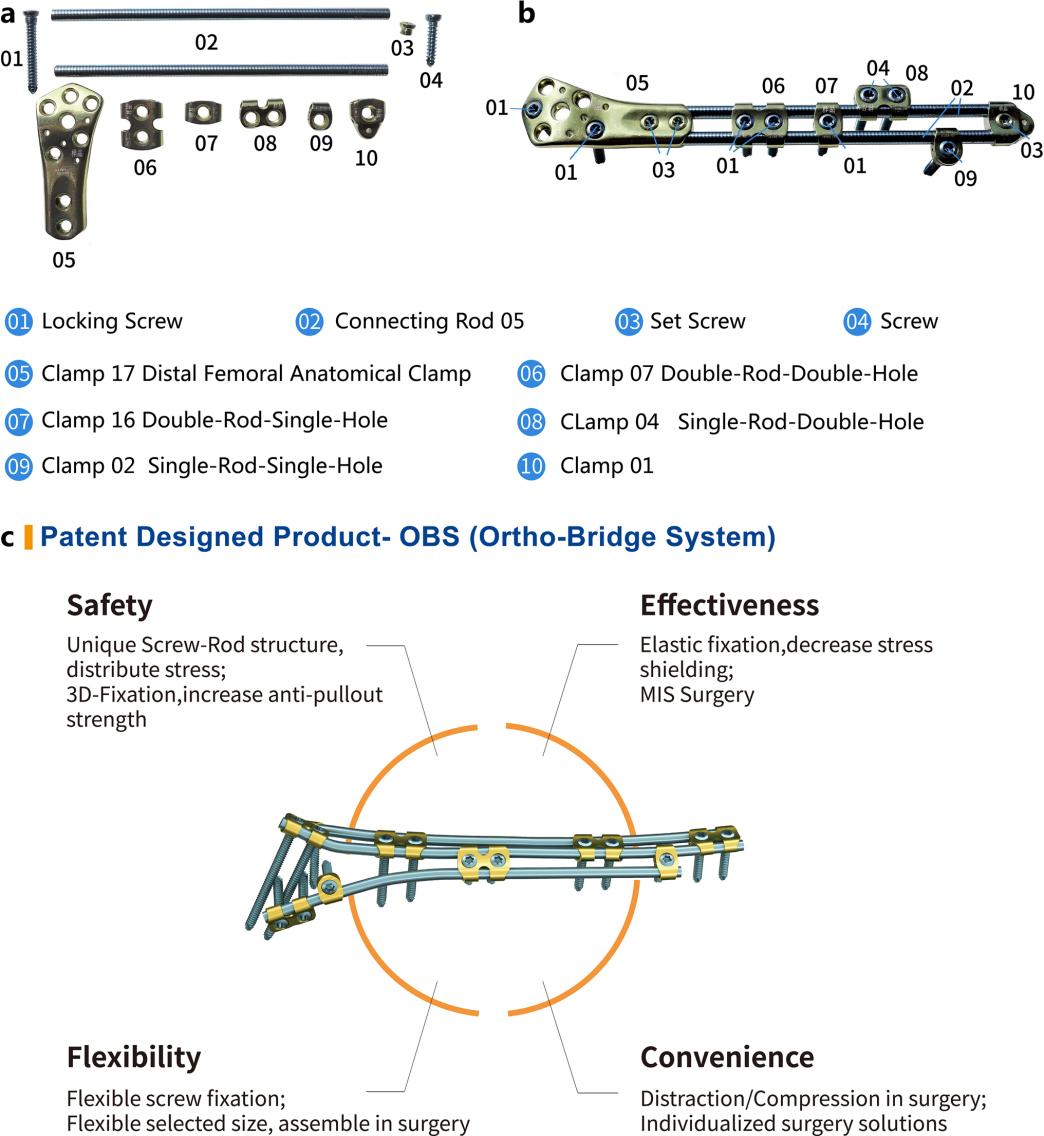
The Ortho-Bridge system**. a** Components of the OBS; **b** schematic diagram of the OBS of the distal femur composed of various components in figure **a**; **c** introduction of specific types of connecting clamp, special clamp, and screws of OBS. Images were selected from the website of Walkman Biomaterial Co., Ltd. *OBS* Ortho-Bridge system

In this article, we introduce another internal fixation instrument for these fractures—the Ortho-Bridge System (OBS). This was developed and designed in China with independent intellectual property rights (Walkman Biomaterial Co., Ltd., Tianjin, China, patent number: ZL200510010654.3), and it consists of different types of single-rod fixation blocks, double-rod fixation blocks, anatomical modules, connecting rods, screws, and locking nuts. The single-rod fixed block is divided into single-rod single-hole block and single-rod double-hole block; double-rod fixed block is divided into double-rod single-hole and double-rod double-hole block; the anatomical blocks are divided into proximal humerus, proximal and distal femur, lateral and medial proximal tibia, and distal tibial fixation block according to the location; the length of the connecting rod can be freely selected and pre-bent shaping; the screws include locking screws and compression screws [9]. The OBS is a new type of fracture fixation device, which incorporates the structural concepts of a locking steel plate, an external fixation bracket, and a spine screw-rod system. The connecting clamp has a rod hole and a locking screw hole, and the fixing rod can rotate and slide freely through the rod hole. The locking screw is double-threaded; the front thread is screwed into the bone and the rear thread is screwed into the locking hole of the connecting clamp. When the rear thread is screwed into the locking hole, the nail tail directly presses on the fixing rod, locking the rod, the clamp, and the nail, thereby forming a locking integral connection. During the operation, the fixed block, anatomical block, and connecting rod can be flexibly selected according to different parts to be freely assembled to complete the fixation of fractures such as cadres or joints. We are in favor of this free combination, and can even complete the full-length fixation of long stem bones such as femur and tibia. According to the fracture situation of each patient, single connecting rod fixation, double connecting rod fixation, or three connecting rod fixation can be designed, which truly realizes personalized fixation (Fig. 1). OBS is suitable for limb and pelvic orthopedics and internal fixation of traumatic fractures. This kind of fixation is an elastic fixation, which can effectively reduce the stress shielding and protect the blood supply of the fractured end, and promote fracture healing [10]. Since 2013, the treatment of simple or complex fractures in limbs, the pelvis, the scapula, and other parts has been performed via this technique, and fracture healing has been achieved. In addition, we found that there was a different process of fracture healing between the OBS and a locking compression plate (LCP). To further study the different healing processes, we reviewed cases that used the OBS for internal fixation of femoral fractures and performed biomechanical analysis of the OBS and LCP. This study aimed to further explain the healing process demonstrated by the application of the OBS in the treatment of simple femoral fractures and analyze its mechanism.

## Materials and Methods

### Clinical Data

This retrospective analysis of existing clinical cases was approved by the institutional review board. There were 11 patients (nine men and two women) enrolled between July 2017 and May 2020. Detailed clinical patient parameters are shown in Table 1. The average age at enrollment was 37.7 years (range 18–54 years). All patients had closed fractures with the following causes of injury: (i) traffic accidents (nine patients) and (ii) falls from a significant height (two patients). Two patients had other fractures with multiple rib fractures and traumatic pneumonia, one had subarachnoid hemorrhage, one had a traumatic renal contusion, and one had traumatic contusions of the liver and spleen. According to the AO Foundation/Orthopaedic Trauma Association classification, there were seven type-A and four type-B femoral shaft fractures. All the patients were diagnosed by clinical symptomatology and radiography.

**Table 1:** Clinical parameters of the patients

No	Sex/age (years)	Injury mechanism	Femoral shaft fracture (AO/OTA Type)	Complicated injury
1	Male, 18	Traffic accident	B2	Renal contusion
2	Male, 34	Traffic accident	A2	
3	Male, 52	Traffic accident	B2	Rib fracture
4	Male, 54	Traffic accident	A3	
5	Female, 45	Traffic accident	A3	
6	Male, 25	Fall from height	B2	
7	Male, 43	Traffic accident	A3	
8	Female, 37	Traffic accident	B2	Subarachnoid hemorrhage
9	Male, 41	Fall from height	A3	Liver and spleen contusion
10	Male, 29	Traffic accident	A3	
11	Male, 37	Traffic accident	B2	Rib fracture

### Preoperative Preparation

After admission, all the patients completed routine blood coagulation, blood biochemistry, and other blood tests. Tibial tubercle traction was performed prior to surgical treatment upon patient stabilization, except for three patients with craniocerebral injury, liver and spleen contusion, and severe hemopneumothorax. The remaining eight patients underwent surgery on the second day after the injury.

### Surgical Procedure

**Fig. 2:**
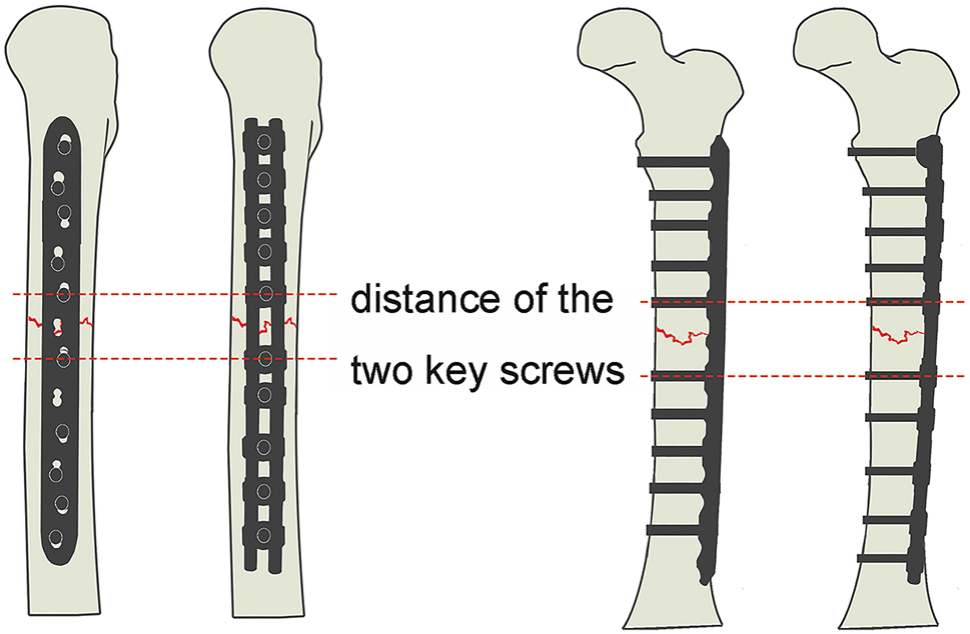
Diagram of LCP and OBS fixation for type-A femoral shaft fracture. The distance between the distal and proximal screws is the key screw distance. *OBS* Ortho-Bridge System, *LCP* locking compression plate

The patient was placed in the supine position with general or epidural anesthesia. The center of the femoral posterolateral approach incision was the femoral fracture site, which was made layer by layer to expose the space and compartment between the lateral femoral muscles. The lateral femoral muscle was pulled up and sharply separated from its attachment point to expose the fracture site without stripping the periosteum. Blood clots were cleared at the fracture site and the soft tissue of the clamp. Longitudinal traction was used to correct the overlapping displacement, and the fracture was reduced and maintained via bone holding forceps. According to the fixation principle that the length of the plate should be 8–10 times that of the fracture area of the simple fracture, the appropriate bridging rod was selected. The sliding module was placed at the distal and proximal ends of the fracture according to the position of the LCP nail holder. Furthermore, key screws were inserted into the proximal and distal nail holes of the adjacent fractures after applying compression forceps. These were screwed into the clamps to tighten the connection rod and clamp, and the final fixation effect is shown in Fig. 2. Anatomical reduction of the femur fractures was confirmed through C-arm fluoroscopy. Finally, the wound was flushed and sutured while placing the drainage tube.

### Postoperative Management

Drainage was removed on the second postoperative day. Weight-bearing function exercise began 1-month post-operation. Weight-bearing was gradually increased with regular radiography review. Once the callus passed through the fracture gap, it could be completely loaded.

### Evaluation Criteria

X-ray examinations were performed every month post-operation to assess fracture healing until a solid continuous callus appeared. Radiographic union was defined as the presence of bridging callus at three of the following four cortices: (i) anteroposterior medial, (ii) lateral, (iii) lateral anterior, and (iv) posterior cortices [11]. Joint function and knee function were measured using the Harris Hip Score (HHS) [12] and Knee Society Score (KSS) [13], respectively, to evaluate subjective and objective quality of life.

### Biomechanical Experiment

*Experimental Materials* The experimental control group used a 12-hole 4.5-mm LCP, which is commonly used for femoral shaft fractures (DePuy Synthes, Raynham, MA). Its unique combination hole is useful for placing common cortical bone screws in the sliding hole depending on the need for sliding compression. It can also be used to place locking screws for fixation, which better reflects the real clinical situation. Tianjin Walkman Biomaterials Co., Ltd. manufactured the OBS, which is composed of connecting rods and fixed blocks. The connecting rod of the experimental group was cut to the same length as the 12-hole LCP.

**Fig. 3:**
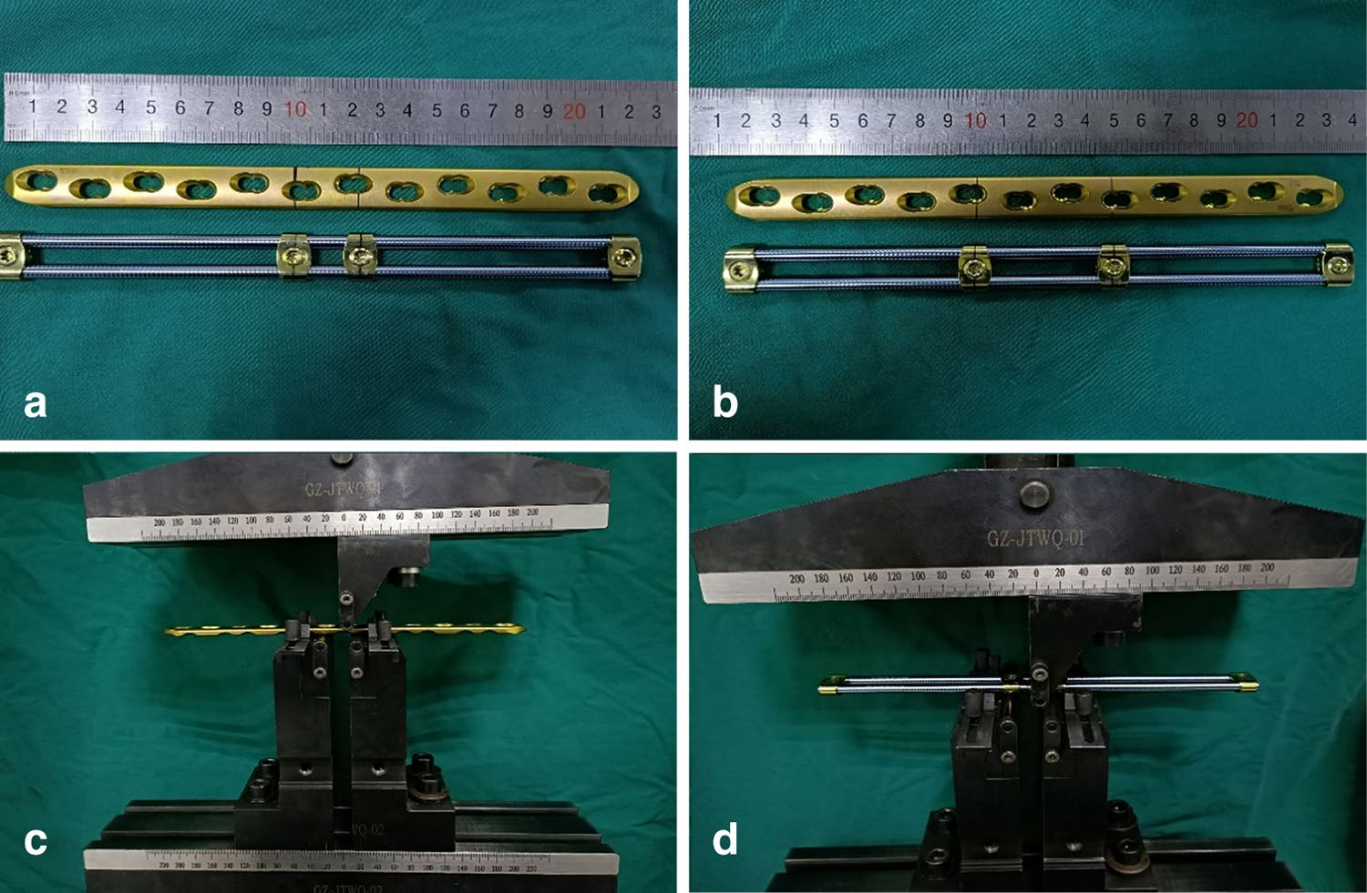
Biomechanics three-point bending test.** a** Two screws are simulated to be placed in the bilateral sliding compression holes of LCP. OBS places screws according to the distribution of LCP screws with a key screw distance of 23 mm; **b** two screws are placed in locking holes on both sides of fracture ends with a distance of two holes from each other, and OBS screw positioning is performed according to the position of the LCP screws with a key screw distance of 49 mm; **c**, **d** static three-point bending test for LCP and OBS, respectively. *OBS* Ortho-Bridge system, *LCP* locking compression plate

*Method* Both OBS and LCPs were randomly divided into four groups, each with six pieces. The LCP group was randomly divided into the following: (i) empty hole group, (ii) empty two-hole group, (iii) empty three-hole group, and (iv) empty four-hole group. The OBS experimental group was set up according to the distance between the two screws, and the sliding module was placed and fixed with the screws in the same position as the LCP group. The distance between the two screws is referred to as the critical screw distance. Each construct was placed in an MTS E45.105 microcomputer-controlled electronic universal testing machine (EA-002; MTS, Eden Prairie, MN) for static three-point bending tests while maintaining temperature at 20 °C. The position of the lower fulcrum of this machine is similar to that of the two key screws of each group, and its midpoint is referred to as the force application point. The initial bending load was 50 N, which was gradually increased to 1000 N at a displacement speed of 2 mm/min (Fig. 3). The sensor is linked to a computer, which displays the displacement and bending force synchronously. The fixed stiffness value (N/mm) was recorded for each group. Stiffness refers to the ability of a material or structure to resist elastic deformation when force is applied. It also characterizes the material’s degree of elastic deformation.

### Statistics

Statistical analysis was completed using Microsoft Excel 2013 (Microsoft, Redmond, WA) and the R statistical environment (R Foundation, Vienna, Austria). The *t* test and *χ*^2^ test were used to compare continuous outcomes and categorical variables, respectively. Statistical significance was established at *P* < 0.05.

## Results

**Fig. 4:**
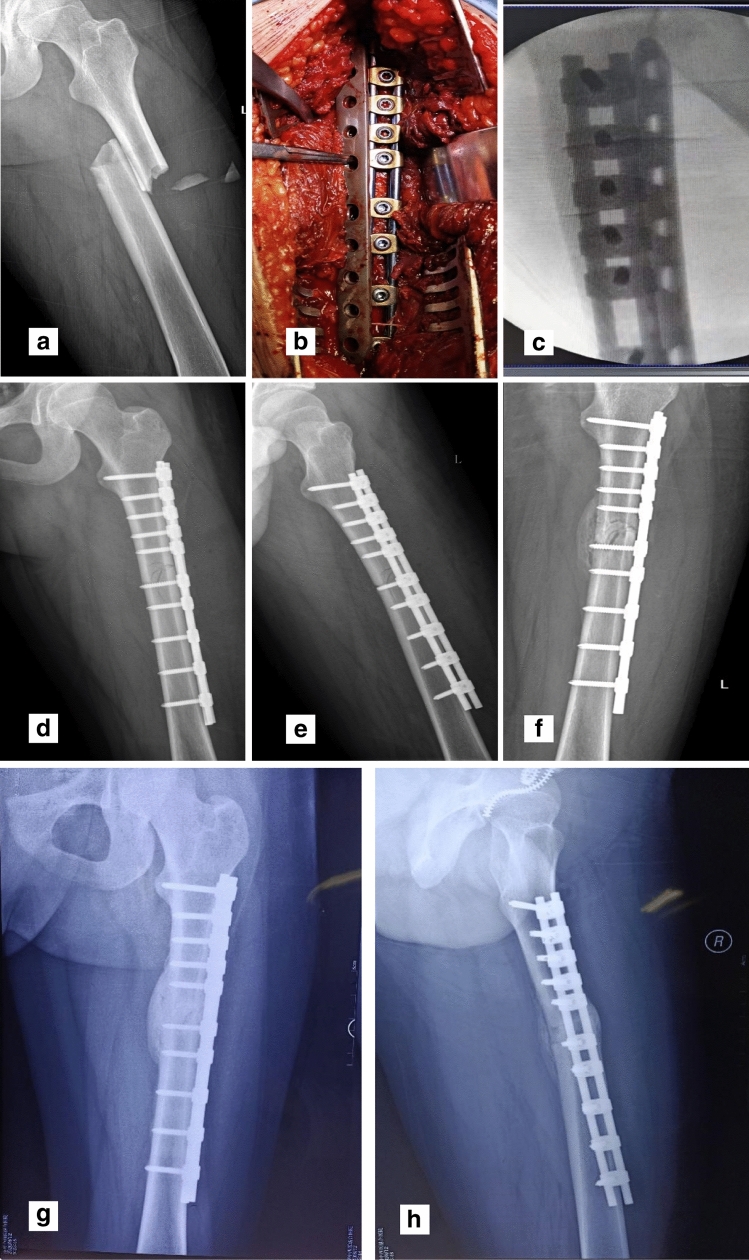
Typical case of a femoral fracture.** a** Preoperative X-ray analysis of B2 fracture of femoral shaft. **b** During the operation, the OBS sliding module is placed, and screws are inserted according to the distribution of nail holes of LCP. **c** Intraoperative C-arm fluoroscopy shows that the distribution of OBS screws is consistent with that of screw holes. **d**, **e** AP and LAT X-ray immediately after operation. **f** One month after the surgery, AP X-ray showed obvious callus growth of the contralateral side of OBS. **g**, **h** AP and LAT X-rays showed fracture healing at 3.5 months after surgery. *OBS* Ortho-Bridge system, *LCP* locking compression plate

The average operation time and blood loss were 82 min (62–106 min) and 252.8 mL (212–326 mL), respectively. After OBS surgery, no complications (i.e., necrosis of incision, infection, or loosening and breakage of the implants) were found. All 11 patients were followed for an average of 12.6 months (8–20 months) and showed excessive callus growth on the contralateral side of the OBS at the first review (1–1.8 months post-surgery). All the patients presented with second fracture healing with an average fracture healing time of 4.5 months (3–5 months). The evaluation of limb function was performed according to the HHS and KSS; nine cases were excellent, and two cases were good. The typical case is shown in Fig. 4 (patient 1).

### Analysis of Static Three-Point Bending Test Results

Once the key screws were placed in the compression holes on both sides of the LCP at the fracture end (distance: 22 mm), a three-point static test was performed. The stiffness of the OBS and LCP was 10,637.7 ± 714.2 and 11,903.5 ± 1047.0 N/mm, respectively. The stiffness of the OBS and LCP was 7709.0 ± 200.1 and 7107.1 ± 263.3 N/mm, respectively, when placed at both ends of the fracture, 1 hole apart (distance: 34 mm). When the key screw distance was 22–34 mm, the stiffness of the OBS was significantly lower than that of the LCP (*P* < 0.05). When the screw was placed at both ends of the fracture, two holes apart (distance: 49 mm), the stiffness of the OBS and LCP was 2946.4 ± 91.3 and 3870.2 ± 67.3 N/mm, respectively. When placed at both ends of the fracture, three holes apart (distance: 66 mm), the stiffness of the OBS and LCP was 2065.3 ± 35.8 and 1494.1 ± 92.2 N/mm, respectively. When the key screw was placed at both ends of the fracture, four holes apart (distance: 82 mm), the stiffness of the OBS and LCP was 750.8 ± 6.3 and 973.9 ± 9.3 N/mm, respectively. According to these results, it can be concluded that the stiffness of the LCP and OBS decreased significantly when the key screw distance was more than two holes (i.e., 49–82 mm), and the LCP was slightly stronger than the OBS (*P* < 0.05) (Table 2).

**Table 2:** Biomechanics three-point bending test data

Stiffness (N/mm)	Bilateral pressor hole group	1-Hole apart group	2-Hole apart group	3-Hole apart group	4-Hole apart group
LCP	11,903.5 ± 1049	7709 ± 600.1	2946.4 ± 91.3	1494.1 ± 92.2	750.8 ± 6.3
OBS	10,637.7 ± 714.2	7107.1 ± 263.3	3870.2 ± 67.3	2065.3 ± 35.8	973.9 ± 9.3
Key screw distance	22 mm	34 mm	49 mm	66 mm	82 mm

## Discussion

In this study, following the treatment of simple femoral shaft fractures with the OBS, we found that the process of fracture healing was different from that of plate fixation. We noticed that the callus grew rapidly, and all patients achieved secondary fracture healing.

Intramedullary nailing fixation is the gold standard of treatment for adult femoral shaft fractures; however, internal plate fixation is particularly advantageous when intramedullary nailing is contraindicated or when there is a lack of surgical experience and image intensifier [14–16]. For patients with head and chest injuries, the best treatment for fractures of the lower limbs is still controversial [17]. Considering the technical difficulty of closed reduction, anatomically reducing fractures and the gaps between fracture fragments cannot be eliminated, especially severe comminuted or segmental fractures, which are still significant challenges for orthopedic surgeons [18, 19]. Locking plates are recommended as they can bridge major fractures and avoid excessive gaps between fragments [20].

The OBS adopts a locking bracket structure with rod connection and a nail–clamp combination, which can evenly distribute stress in the working area through the elastic and stress conduction characteristics of the rod to effectively alleviate the stress concentration and shielding, reducing the rupture risk of the internal fixation, and achieving a micro-motion effect of the fracture end. The OBS does not require, or rarely requires, stripping of the periosteum to fix the fracture. The OBS is like a built-in external fixator, which directly spans the fracture site and avoids compression of the periosteum and cortical bone. In the case of weight-bearing, this technique is in a state of continuous and dynamic compression, and the longitudinal compressive stress stimulates the formation of callus to avoid stress shielding and prevent osteoporosis. The healing time and healing process of OBS and femoral intramedullary nail are basically similar [21, 22]. The healing time of OBS for femoral shaft fractures was not significantly different from that of intramedullary nailing, and it was not inferior to intramedullary nailing for femoral shaft fractures.

The size of the fracture fragment gap and the initial stability of the fracture site are key factors that determine the type of healing (i.e., primary or secondary) and time of recovery. The stiffness of the fixation structure is the main determinant of the movement of the fracture site, thus affecting the mechanism and progress of fracture healing. Therefore, fracture fixation should follow the following principles: (i) if secondary healing is the purpose of simple fracture healing, the movement of fragments along the axial direction is conducive to the formation of cartilage callus [23], and (ii) the gap and movement amplitude should be kept small (range 0.2–1 mm; fracture gap < 2 mm). Other fracture modes (i.e., spiral fracture and multiple fragments) can withstand higher strain amplitudes.

Primary healing occurs once the fracture is stable and refers to intramembrane ossification, which is the direct transition of mesenchymal cells to osteoblasts [24]. Type A and B femoral fractures were fixed with the OBS, and excessive callus formation was found on the contralateral side of the internal fixator. This was quite different from plate fixation, which piqued our curiosity. Therefore, OBS fixation was performed in suitable patients with simple femoral fracture, and the sliding module of the OBS was screwed according to the layout of plates and screws. All patients presented with excessive contralateral callus growth. Thus, we conducted the biomechanical static three-point bending test to compare the biomechanics of the OBS and LCPs. The results showed that the OBS was less stiff than the LCP when the distance between the compression screw hole and the remaining hole is selected at both ends of the fracture. Specifically, the key screw distance (i.e., the distance between the screws less proximal and distal to the fracture) of the OBS was 3 ± 1 cm, which was significantly different from that of the LCP. The OBS was significantly more elastic than the LCP when using this distance. OBS application for fracture fixation was relatively stable. In the early stage of fracture fixation, axial stimulation and mechanical movement of the fracture end would be produced during partial load bearing, which stimulates callus growth and fracture healing. During the follow-up period, abundant callus formed during OBS fracture fixation according to the principle of relative stability. Due to the plate-screw connection’s rigidity, the locking plate produced less bone stress, which may inhibit interfragmentary movement to a range inappropriate for optimal indirect fracture healing. The stiffness of the fixation construct is the principal determinant of fracture site movement, which mainly affects the mechanism and progress of fracture healing [25, 26]. This can be achieved by applying axial micro-movements on the implant and changing the mechanical strain on healing tissues [27]. When a simple fracture is fixed with the OBS, its stiffness is lower than that of the LCP within a certain distance of the key screw. This can also produce axial micro-motion to stimulate fracture healing and lead to secondary healing with rich callus growth.

However, several limitations are acknowledged in this study. The study is a single-center retrospective case study, the sample number is relatively small, and with a short follow-up time, thus, the results of this study may not adequately reveal the characteristics of OBS in the treatment of femoral fractures. In future research, with the continuous improvement of patients' recognition, there will be more and more cases, which can better improve the clinical efficacy of OBS in the treatment of femoral fractures.

## Conclusion

Applying OBS at the femoral shaft fracture site reduced the time for bone consolidation and promoted earlier and tri-plane uniform callus formation that could indicate faster reconstruction of the fracture site. This is particularly interesting for simple fracture patterns. Thus far, these fracture patterns are completely different from the healing process of LCP construct fixation. These are instances of secondary fracture healing or other healing patterns, which necessitate further investigation.

## Data Availability

The datasets generated during and/or analyzed during the current study are available from the corresponding author on reasonable request.
